# Procedural sedation in pediatric dentistry: a narrative review

**DOI:** 10.3389/fmed.2023.1186823

**Published:** 2023-04-26

**Authors:** Feng Gao, Yujia Wu

**Affiliations:** ^1^Department of Anesthesiology, Stomatological Hospital of Chongqing Medical University, Chongqing, China; ^2^Chongqing Key Laboratory of Laboratory of Oral Diseases and Biomediacal Sciences, Chongqing, China; ^3^Chongqing Municipal Key Laboratory of Oral Biomedical Engineering of Higher Education, Chongqing, China

**Keywords:** procedural sedation, pediatric dentistry, behavior management technology, drug delivery, medications

## Abstract

Procedural sedation and analgesia are now considered standard care for managing pain and anxiety in pediatric dental patients undergoing diagnostic and therapeutic procedures outside of the operating room. Anxiolysis, which combines both pharmacologic and non-pharmacologic approaches, plays a significant role in procedural sedation. Non-pharmacologic interventions such as Behavior Management Technology can help reduce preprocedural agitation, ease the transition to sedation, reduce the required amount of medication for effective sedation, and decrease the occurrence of adverse events. As the introduction of novel sedative regimen and methods in pediatric dentistry, the potential role of mainstay sedatives administered by new routes, for new indications, and with new delivery techniques, should be considered. The purpose of this paper is to examine and discuss the current state of sedation techniques in pediatric dentistry.

## 1. Introduction

Dental phobia and dental anxiety are both terms used to describe fear and anxiety related to dental procedures, but they differ in their severity. Dental phobia refers to an enduring and excessive fear of dental stimuli and procedures that results in avoidance or significant distress. Dental anxiety, on the other hand, is a heightened fear of dental procedures that may or may not meet the complete criteria for a diagnosis of phobia. Children and adolescents who suffer from odontophobia or dental anxiety may exhibit disruptive behaviors during examinations and treatment, ranging from restlessness to full-blown tantrums (1); In the most extreme cases, young individuals with dental anxiety may refuse treatment, even when they are experiencing significant pain that could be relieved with proper care (2). Prevalence estimates of dental anxiety in youth are somewhat variable, with estimates ranging from around 5 to 20%. However, this is likely to be an underestimate in the general population since children and adolescents with the most severe dental anxiety may avoid dental treatment entirely or seek care only at specialty clinics (3). Dental anxiety and fear vary across a continuum from very mild anxiety and fear to severe and debilitating dental phobia (4). A child’s capability to regulate their own behavior and cooperate during a procedure relies on their chronological age as well as their cognitive and emotional development. Children who have low or moderate levels of fear or anxiety can be effectively managed by establishing a trusting relationship, utilizing good communication skills, showing empathy, providing careful treatment, and using some basic non-pharmacological techniques. Conversely, highly anxious/fearful or phobic children may necessitate targeted pharmacological support in addition to the utilization of behavior guidance strategies, such as behavioral guidance techniques, nitrous oxide sedation, intravenous sedation, and general anesthesia (5). The selection of sedative agents and approach is typically influenced by factors such as the type of procedure, the patient’s comorbidities and temperament, and the clinician’s preference. The primary objectives of sedation usually include providing anxiolysis, analgesia, amnesia, safety, efficacy, and the ability to facilitate the completion of the procedure (6). Numerous short procedures may be conducted using distraction and guided imagery techniques in conjunction with the application of topical or local anesthetics, and minimal sedation if necessary (7). However, lengthier procedures that demand immobility involving children younger than six years or those with developmental delays often demand a greater level of sedation to gain control of their behavior (8).

A dental practice environment presents several additional challenges, such as the use of the low-speed drill, continuous vibration, constant suction, and the administration of local anesthesia injections. All of these concurrent stimuli may cause the child to remain in a heightened state of alertness (9). Because of the significant levels of anxiety and fear experienced by young children during dental procedures, conventional non-pharmacological methods are often considered inadequate (10). Due to dental fear and/or dental behavior management problems, some children may not be able to cooperate for treatment using local anesthesia and psychological support alone, and passive restraint was ranked as the least desirable technique. Parents have reported concerns that protective stabilization may increase their child’s fear and be stressful for them (11). Although treatment can be performed under general anesthesia, it is generally recommended to avoid it whenever possible due to the need for specialized resources and the potential risks, including the risk of death (12).

## 2. Definition of procedural sedation

The International Committee for the Advancement of Procedural Sedation provides the following definition for the practice of procedural sedation: The practice of procedural sedation is the administration of one or more pharmacological agents to facilitate a diagnostic or therapeutic procedure while targeting a state during which airway patency, spontaneous respiration, protective airway reflexes, and hemodynamic stability are preserved, while alleviating anxiety and pain (13). In order to meet the need for pain control, analgesics can be combined with sedative agents, a technique referred to as procedural sedation analgesia (PSA) (14). Procedural sedation is categorized as a state of minimal or moderate sedation in accordance with the American Society of Anesthesiologists (ASA) classification (15). Various evaluation methods have been developed to determine the degree of sedation, with the Ramsey scale being the most commonly used. This scale scores on eight characteristics, with scores indicating anxiolysis (2 to 3), moderate sedation (4 to 5), deep sedation (6), and general anesthesia (7 to 8) (16) (Table 1).

**Table 1:** Terminology to describe levels of procedural sedation.

Level of sedation	Description
Minimal	Also called *anxiolysis*; the patient is awake and relaxed, and is able to respond normally to verbal stimuli.
Moderate	Also called *conscious sedation*, the patient experiences a state of depressed consciousness while remaining responsive to verbal requests or tactile stimuli. Spontaneous breathing remains unimpaired, and no respiratory support is required.
Deep	The patient displays a decreased level of arousal but responds intentionally to painful stimulation, potentially requiring assistance to maintain airway patency and adequate ventilation. Cardiovascular function is usually maintained.
Dissociative	The patient is in a trance-like and cataleptic state, experiencing deep analgesia and anesthesia. Despite this, the patient retains protective airway reflexes, spontaneous ventilation, and cardiovascular function.

Choosing the minimal number of medications and ensuring that the drug selection aligns with the type and objectives of the procedure are crucial for safe practice. Dental sedation is unique in that it remains, along with emergency medicine, the area of procedural sedation where the proceduralist can also be supervising the administration of sedation (17).

The perfect sedative substance would alleviate anxiety and enhance conduct, thus facilitating the execution of dental procedures and offering a pleasant experience for the patient. It ought to be administered safely in the primary care sector and possess a generous margin of safety (12). The purpose of this paper is to discuss the current state of pediatric dental sedation.

## 3. Goals and safety of procedural sedation

As per the American Academy of Pediatric Dentistry (AAPD) and the American Association of Pediatrics (AAP),the objectives of sedation encompass: (1) to guard the patient’s safety and welfare; (2) to minimize physical discomfort and pain; (3) control anxiety, minimize psychological trauma, and maximize the potential for amnesia; (4) to modify behavior and/or movement to allow the safe completion of the procedure; and (5) to return the patient to a state in which discharge from medical/dental supervision is safe, as determined by recognized criterial (8). All of these objectives must be attained while ensuring that the patient retains airway control, oxygenation, and hemodynamic stability. The endeavors of the Pediatric Sedation Research Consortium have significantly enhanced our understanding of procedural sedation, and have demonstrated the remarkable safety of procedural sedation when administered by proficient and enthusiastic practitioners from various disciplines, employing the aforementioned modalities and skills that prioritize a culture of sedation safety. Nonetheless, these pioneering investigations also reveal a persistent but low incidence of potential life-threatening events induced by sedation, such as apnea, airway obstruction, laryngospasm, pulmonary aspiration, desaturation, and others, even when administered by a dedicated team of specialists. These studies have helped to establish the essential competencies required to rescue children experiencing adverse events while under sedation (8). Consequently, the provider must possess a thorough understanding of the available pharmacologic agents to administer the most appropriate medication required for a specific procedure at the lowest dose and highest therapeutic index. Furthermore, for each pharmacologic agent selected, the medical professional must be aware of the drug’s peak response, onset, and duration of action (18).

## 4. Patient selection and assessment

Patient evaluation should encompass a comprehensive medical, dental, and social history. The American Society of Anesthesiologists Physical Status (ASA-PS) classification is an appraisal performed by an anesthesia provider prior to anesthesia administration, with the sole objective of evaluating the patient’s physical condition (18). Patients classified as ASA Class I or Class II may be regarded as suitable candidates for outpatient conscious sedation. Patients in ASA Class III and Class IV present unique challenges necessitating personalized consideration and are optimally managed in a hospital setting (19).

## 5. Selection of procedural sedation intervention in children

Sedation exists on a continuum, and the physiologic effects may vary significantly depending on various factors, including the medication, dosage, delivery route, and patient characteristics (20). When selecting pharmacologic or non-pharmacologic interventions for sedation, the child’s developmental status, clinical circumstances, and overall condition must be taken into account (21). The invasiveness of a medical procedure may affect the perceived degree of sedation. As the invasiveness of a procedure increases, a deeper level of sedation is typically required, necessitating higher doses of medication and potentially increasing the risk of adverse events during or after the procedure. The expected duration of the procedure is another essential factor to consider, in addition to invasiveness. A more extended procedure will necessitate a greater quantity of sedative drugs than a shorter intervention (22). Almost all non-dissociative drugs used for procedural sedation and analgesia can induce a state of general anesthesia, resulting in the loss of protective airway reflexes. Therefore, continuous monitoring is crucial, and clinicians must be prepared to rescue patients from levels of sedation deeper than intended (23). Achieving adequate sedation requires both anxiety reduction and pain control, making excellent local anesthesia critical (24). Guidelines for procedural sedation in the USA and Europe suggest selecting an appropriate sedative agent based on the procedure and patient characteristics or for its ease of dosing to achieve and maintain sedation while minimizing adverse events (25, 26).

## 6. Behavior management technology

Ideally, only children who suffer from high dental anxiety or fear, or those with diagnosed dental phobia, should be referred to general anesthesia. The National Consensus Development Conference on Anesthesia and Sedation in the Dental Office recognizes that “behavioral approaches are often overlooked as effective mechanisms for relieving patient apprehension” and suggests that sedation and general anesthesia may be unnecessary in situations when psychological and behavioral approaches are effective (27). Non-pharmacologic interventions, such as behavioral and cognitive approaches, may prevent the need for procedural sedation in many children. Their use has been shown to ease the transition into a state of sedation, reduce the amount of medication required, and subsequently, the depth of sedation, and decrease the frequency of adverse events (28). The American Academy of pediatric Dentistry (AAPD) recommended concentrating more on non-pharmacologic intervention in future studies (29). To date, there are many behavior management techniques (BMTs) available to dental practitioners, including tell-show-do (TSD), relaxation, distraction, systematic desensitization, modeling, audio analgesia, hypnosis, and behavior rehearsal. Among these, TSD and modeling are the most commonly used BMTs by pediatric dentists (30). Two literature reviews have suggested that distraction techniques are effective in reducing anxiety and pain during dental procedures, but the level of evidence supporting this is low (31, 32). The effectiveness of listening to music during dental procedures in reducing anxiety and pain is not clear, as there are conflicting results in the literature. Some studies suggest that music can be helpful in reducing anxiety and pain, while others do not find significant benefits (33). The use of virtual reality headsets to provide a calming and distraction-inducing environment has shown promising results in reducing anxiety and stress levels in patients undergoing dental procedures. This technology works by creating an immersive environment that distracts the patient from the dental procedure, which can help to decrease pain and anxiety levels (34, 35). Communication with parents or legal guardians is crucial for effective guidance of a child’s behavior during dental procedures. Maintaining open communication with parents can help ensure the child’s safety and comfort during the procedure, and it can also help alleviate any anxiety or concerns that the parents may have (36) (Table 2).

**Table 2:** Brief description of different BMTs.

Technique	Brief description
Tell-show-do	The tell-show-do technique involves verbal explanations of procedures in phrases appropriate to the developmental level of the patient (tell), followed by demonstrations of the visual, auditory, olfactory, and tactile aspects of the procedure in a non-threatening setting (show), and finally, completion of the procedure without deviating from the explanation and demonstration (do)
Positive rein-forcement	an efficacious method for rewarding desired behaviors and reinforcing their recurrence
Nonverbal communication	Nonverbal communication entails reinforcing and guiding behavior through suitable physical contact, posture, facial expression, and body language
Effective communication	It is vital in establishing a relationship with the child and fostering a positive attitude toward dental health
Modeling	Modeling is based on the psychological principle that children learn by observing the behavior of others. Therefore, by modeling appropriate behavior, the dentist can capture the child’s attention, promote compliance, and prevent negative attitudes or behaviors
Voice control	Voice control refers to the intentional modification of voice volume, tone, or pace to influence and guide the patient’s behavior
Parental separation	Involves utilizing the presence or absence of parents to obtain cooperation for treatment
Distraction	Distraction is a technique used to redirect the patient’s attention from a potentially unpleasant procedure
Hand-over-mouth technique	The technique of hand-over-mouth is employed to redirect inappropriate behavior that cannot be modified by basic behavioral management techniques. In this technique, the dentist gently places their hand over the child’s mouth while calmly explaining the behavioral expectations, all while ensuring that the child’s airways remain open
Protective stabilization	Partial or complete immobilization of the child may be necessary to protect them from injury, particularly in cases where the child is uncooperative or disabled
Hypnosis	Hypnotic induction may be utilized to help relax a child during dental procedures, as it has been shown to be effective in reducing anxiety in children

Research indicates that modern parents may be less tolerant of physical and attentional behavior guidance than previous generations, and may be more inclined to accept or request procedural sedation or general anesthesia for their child’s dental treatment (37). As a result, pharmacological behavior guidance is now commonly employed in the dental profession (38). The use of pediatric sedation outside of the operating room is a growing trend in the field of anesthesiology. However, few new sedatives have been introduced in the last decade, highlighting the need for further development of new routes and methods for delivering existing anesthetic agents (39).

## 7. The method of drug delivery

### 7.1. PO

In the pediatric setting, it is generally advised to circumvent aversive routes of administration, such as intravenous (IV) administration, and instead opt for the oral route of administration. Among pediatric dentists, oral sedation is the preferred and most commonly utilized method of administration (24). Oral sedation is not only cost-effective but also straightforward to administer, and is generally well-received by most children. Importantly, it does not require injection or cannula insertion, which adds to its appeal as a safe and convenient method of sedation (40). Oral medications are particularly well-suited for inducing minimal to moderate sedation in the dental setting. While these medications can lead to mild impairment of cognitive function and coordination, they do not typically affect ventilatory or cardiovascular functions (41). When drugs are administered orally, they undergo significant reduction in concentration due to hepatic first-pass metabolism. As a result, oral sedation may have certain drawbacks, such as the inability to titrate the dose to achieve the desired effect, as well as the need for a single-bolus dosing regimen (8).

### 7.2. Intranasal and inhalation sedation

One of the major advantages of transmucosal administration is that it allows for direct absorption of drugs into the systemic circulation, bypassing hepatic first-pass metabolism and resulting in increased bioavailability and faster onset of action compared to oral sedation. In addition, transmucosal administration typically causes less discomfort than intravenous sedation, making it a more favorable option for patients (42). The extensively vascularized nasal mucosa and the olfactory tissue in direct proximity to the central nervous system expedite swift transportation into the bloodstream and brain, with onsets of action comparable to that of intravenous therapy (43). Despite its simplicity, relative painlessness, and the need for less patient cooperation, intranasal administration has been linked with mucosal irritation (44). When comparing the administration of intranasal midazolam *via* drops and aerosolized forms, aerosolization was better tolerated and resulted in less aversive behavior (45). In dentistry, the intranasal route is regarded as parenteral and hence, may necessitate a more comprehensive sedation license.

### 7.3. Intravenous sedation

Intravenous administration is the swiftest way for a drug to take effect and the optimal method for titrating a drug to achieve a specific blood concentration. Nonetheless, a significant drawback of IV administration, particularly in pediatric dental procedural sedation, is the requirement for continuous venous access and the associated puncturing of the vein (46).

## 8. Anesthesia technology

### 8.1. Total intravenous anesthesia

Total intravenous anesthesia (TIVA) is a general anesthesia technique that employs a blend of intravenous anesthetics without the administration of any inhalation anesthetics. The primary objectives of this approach are to achieve a seamless induction and safe maintenance of anesthesia, along with swift emergence. Over the last few years, TIVA has gained immense popularity among pediatric anesthesiologists (47). In contrast to inhalation anesthetics, Lauder et al. reported several advantages of TIVA during anesthesia in pediatric patients. According to their findings, significant reductions in laryngospasm, nausea/vomiting, emergence delirium, airway reactivity, stress hormone release, and pain were observed in pediatric patients undergoing TIVA (48). In the case of TIVA, it is necessary to establish IV access before administering IV drugs. However, most children worldwide dread the thought of an ‘IV’. To alleviate this fear, anesthesia is commonly induced *via* a mask. Once an appropriate depth of anesthesia has been attained, IV access can then be obtained (49).

### 8.2. New techniques for sedation delivery

Target-controlled infusions (TCI) have the potential to become the future of pediatric sedation. Advancements in computer technology, pharmacokinetic modeling, and IV infusion delivery devices have facilitated the development of TCI. TCI devices deliver a bolus, followed by exponentially declining infusions to quickly achieve and sustain a stable drug concentration in the plasma or at the site of drug effect. In pediatrics, a significant challenge for TCI is identifying models that are most suitable for children across various age ranges (50). Although there is insufficient evidence to provide definitive recommendations regarding the use of TCI versus MCI (manually controlled infusion) in clinical anesthesia practice, it has been reported that the use of TCI led to fewer interventions than MCI. This discovery provides impetus for further research to develop pediatric models for TCI and assess their practical applicability (51).

A more recent development in this field is the concept of modifying TCI to a ‘closed-loop system.’ Closed-loop delivery systems provide the advantage of giving feedback to the delivery system, which can then adjust the delivery. Various procedures require different depths of sedation, and each procedure has different demands for the level of sedation over its duration. With precise pharmacokinetic and pharmacodynamic studies tailored to different procedures, it may be feasible to target TCI and closed-loop delivery more effectively to the procedure (52) (Table 3).

**Table 3:** Characteristics of several types of sedation.

	Advantages	Disadvantages
PO	Economical, easily manageable from a technical standpoint, widely embraced by the majority of children, and devoid of the need for injections or cannula insertion.	Decrease in concentration resulting from hepatic first-pass metabolism; inadequate ability to adjust the dosage to achieve desired effects, reliance on a single bolus administration, and notable inter-individual variations.
Intranasal (IN) and inhaled routes	Prevention of hepatic first-pass metabolism, leading to lower levels of discomfort compared to intravenous sedation and greater acceptance among patients.	Mucosal irritation; may require a more comprehensive sedation license.
Intravenous (IV), TIVA, TCI and ‘closed-loop system’	The most rapid method for a drug to produce an effect and the optimal approach for tailoring drug dosage to achieve a specific blood concentration.	Significant investment in both personnel and equipment, the requirement for continuous venous access, and the associated venipuncture procedure.

## 9. Medications

### 9.1. Nitrous oxide

Nitrous oxide is one of the top choices for mild sedation during dental procedures (53). For numerous years, a combination of nitrous oxide and oxygen, featuring diverse concentrations, has been efficaciously utilized to furnish analgesia during various painful procedures in children (54). The utilization of nitrous oxide for minimal sedation entails the delivery of nitrous oxide at concentrations of ≤50%, blended with oxygen, and without any concurrent administration of other sedatives, opioids, or depressant medications, to an otherwise healthy patient belonging to ASA class I or II. During the procedure, the patient retains the capacity for verbal communication (55). Research investigating nitrous oxide as a sole therapeutic agent has demonstrated that dental procedures were accomplished in 52% of cases with a 40% concentration, and up to 85% with an equimolar combination, with no reported adverse effects (56, 57). According to a systematic review and meta-analysis, the estimated efficacy rates of nitrous oxide-oxygen procedural sedation in pediatric populations was 91.9% (95% CI:82.5 ~ 98.2%) (58). The effectiveness of nitrous oxide-oxygen procedural sedation is diminished in cases of severe anxiety or fear. Additionally, due to their inherently uncooperative nature, children may not readily accept the nasal mask or may exhibit uncontrolled movements during the initial stages of sedation (59). Considering its significant diffusibility, administration of nitrous oxide ought to be avoided in patients who have the potential for closed-space diseases, such as bowel obstruction, middle ear disease, pneumothorax, or pneumocephalus.

### 9.2. Midazolam

Midazolam is a short-acting benzodiazepine that rapidly produces anxiolytic, sedative, hypnotic, anticonvulsant, and muscle relaxant effects, and often leads to anterograde amnesia. The drug binds to the benzodiazepine receptor in the central nervous system (CNS) and augments the inhibitory effects of the neurotransmitter gamma-aminobutyric acid (GABA). The inhibitory effects of GABA are generated by augmenting the influx of chloride ions through the nerve cell’s ion channels, thereby decreasing the cell’s capacity to initiate an action potential (60). The reliable and consistent sedative and amnestic effects of benzodiazepines render them an appealing class of drugs for utilization in pediatric procedural sedation (61). Benzodiazepine sedatives, with midazolam being regarded as the standard of care, have been used extensively for procedural sedation in pediatric patients (26).

Midazolam is a frequently used sedative agent in pediatric dentistry due to its swift sedative action, anxiolytic properties, and amnestic effects (62). The European Association of Pediatric Dentistry (EAPD) recommends the use of oral midazolam for sedation of children requiring dental treatment. The administration of oral midazolam for dental sedation in pediatric patients is supported by moderate-quality evidence and is deemed safe at appropriate dosages while being well-tolerated by children (63). However, there have been reports of paradoxical reactions in a small number of cases, such as hyperactivity, aggressive behavior, inconsolable crying, and psychomotor disorders. These reactions are typically mild and self-limiting, but healthcare providers should be aware of the possibility and monitor patients closely during and after the administration of midazolam (64). Oral sedation with midazolam does not allow for the same degree of titration as intravenous methods, which makes it important to exercise caution when anticipating potential pharmacodynamic interactions with other drugs. For example, combining midazolam with other sedative drugs, such as opioids or barbiturates, can lead to excessive sedation or respiratory depression. Similarly, the use of midazolam with antipsychotics, H1 antihistamines, or centrally acting antihypertensive drugs can result in additive sedative effects, which may increase the risk of adverse events. Therefore, it is essential to review a patient’s medical history and medication profile before administering midazolam or any other sedative medication (65). Flumazenil can reverse respiratory depression or apnea and paradoxical reactions. However, it should not be administered to patients with seizure disorders or those receiving chronic benzodiazepine treatment due to the potential risk of precipitating seizures or withdrawal symptoms. The oral route of midazolam administration (PO) is preferable, particularly in children, as it is less traumatic. Oral midazolam can be administered 20 to 30 min before the procedure. The standard dosage of oral midazolam for moderate sedation in children typically ranges from 0.25 to 1 mg/kg (66).

### 9.3. Ketamine

Ketamine is a noncompetitive antagonist of the N-methyl-D-aspartate receptor that impedes the discharge of the excitatory neurotransmitter glutamate. It exerts its anesthetic, amnesic, and analgesic effects by lowering central sensitization and the “wind-up” phenomenon. Due to its efficacy and widespread use, Ketamine is a popular choice for painful procedures (67). At present, Ketamine is the solitary dissociative sedative agent employed in clinical practice. It can be utilized as a sole pharmacological intervention for painful procedures.

When administering procedural sedation to pediatric patients, Ketamine can be administered *via* various routes such as intravenous, intramuscular, and intranasal. The use of Ketamine, either alone or in combination with other agents, can safely, effectively, and promptly induce sedation in pediatric patients, regardless of the chosen route of administration (68). There exists a “dissociative threshold” of roughly 1–1.5 mg/kg intravenously or 3–4 mg/kg intramuscularly for Ketamine, beyond which increasing dosages do not lead to heightened effects. Horizontal nystagmus is a characteristic outcome of Ketamine administration, and parents should be apprised that this is a normal consequence of Ketamine use to avoid undue anxiety.

Esketamine, a dextrorotatory enantiomer of Ketamine, has a lower incidence of psychotropic side effects than racemic Ketamine. This leads to lesser impairment in concentration capacity and primary memory, as well as faster recovery. Esketamine has the potential clinical advantage of a shorter recovery time and quicker orientation recovery time compared to racemic Ketamine (69). It can be given in a variety of ways, including nasal administration.

### 9.4. Dexmedetomidine

Dexmedetomidine (DEX) is a selective alpha-2 adrenergic receptor agonist that can be employed for pediatric sedation *via* intranasal, oral, or buccal routes of administration (70). There has been an upward trend in the utilization of dexmedetomidine, particularly *via* the intranasal route. Unlike other sedative agents, DEX does not interact with opioid and GABA receptors, thereby averting respiratory depression. Its capacity to maintain spontaneous ventilation, spare respiratory effects, and uphold upper airway tone renders DEX an appealing option for procedural sedation in children, particularly those who are susceptible to apnea, hypoventilation, or respiratory depression (71). DEX is an exceptional drug when the primary objective is to achieve sedation and immobility in children. It can be employed as a safe and effective option and is the preferred drug for inducing sedation in diagnostic imaging procedures (72).

Intranasal DEX helps overcome the challenge of obtaining intravenous access in pediatric patients who are undergoing various diagnostic studies. This practice is becoming increasingly popular among sedation providers outside of the operating room owing to the reduction in emotional stress that children experience with the intranasal administration route (73). The effects of DEX resemble those of natural sleep, and it is known to be a safe and neuroprotective agent in anesthetic neurotoxicity (74, 75). DEX has a slightly longer onset time (15 to 30 min) and a more prolonged duration of action (55 to 100 min) when compared to midazolam (76). Dexmedetomidine is an excellent sedative premedication option for uncooperative children and can be utilized as a sole agent for sedation. However, current evidence suggests that it may not offer significant benefits when routinely administered as an adjunct to general anesthesia in children undergoing simple day case procedures (77). As Lee-Archer et al. comment, their current standard of care without dexmedetomidine is appropriate and no change in practice is needed. Until, or unless, further larger trials are performed there is no reason to expose children to unnecessary additional drug exposure when there is no clear evidence of its efficacy in these clinical situations.

### 9.5. Fentanyl

Fentanyl is a potent and highly selective opioid agonist with a rapid onset and short duration of action. Unlike other opioids, it lacks histamine release and has fewer cardiovascular effects. It is primarily used for immediate relief of severe pain. The nasal spray formulation of fentanyl is a safe alternative and eliminates the need for needle use (78). However, the safety profile of oral transmucosal fentanyl citrate is poor, with complications in up to 46 percent of patients and a high rate of emesis (79). This has led to the recommendation that it not be used for procedural sedation (80).

Alfentanil is a synthetic, short-acting μ-opioid agonist that is associated with fewer adverse events, including less respiratory depression and postoperative nausea and vomiting (PONV), than fentanyl. When compared to fentanyl, alfentanil has a shorter half-life and faster recovery time, which provides significant clinical advantages during outpatient anesthesia (81).

### 9.6. Propofol

Propofol has been a revolutionary anesthetic agent ever since its inception four decades ago, and is still regarded as a nearly perfect anesthetic agent. Its outstanding performance in clinical settings can be attributed to its prompt onset, brief duration of action, and negligible adverse effects (82). Sub-anesthetic dosages of propofol administered through intravenous conscious sedation infusion have eased dental procedures for apprehensive children. Moreover, it can be injected toward the conclusion of an examination or procedure to mitigate the occurrence and intensity of emergence agitation (83). However, intravenous propofol induction remains problematic due to the challenges involved in obtaining vascular access in distressed and alert children (84). The most critical adverse effect of propofol is its potent respiratory depression, which may lead to sudden apnea.

### 9.7. Etomidate

Etomidate is an imidazole-based agonist of the γ-aminobutyric acid type A (GABA_A_) receptor used for the induction of general anesthesia and sedation. It produces a rapid onset of hypnotic effect similar to barbiturates and propofol but does not possess any analgesic properties. A notable advantage of etomidate is its minimal impact on the cardiovascular system. It causes negligible systemic changes in blood pressure and heart rate, making it an ideal drug for patients who are hemodynamically unstable. Additionally, etomidate causes minimal respiratory depression and does not trigger histamine release, rendering it a highly favorable agent (85). Etomidate may cause adrenocortical suppression by inhibiting the cytochrome P450 enzyme 11β-hydroxylase, which renders it unsuitable for use as a maintenance drug for anesthesia or sedation. Consequently, etomidate is primarily reserved for inducing anesthesia in patients who are hemodynamically unstable (86).

### 9.8. Chloral hydrate

Chloral hydrate is a non-opiate, non-benzodiazepine sedative-hypnotic drug. Although the liquid formulation of chloral hydrate is no longer available commercially, some hospital pharmacies are now compounding their own formulations. In pediatric dental practice, low-dose chloral hydrate (10–25 mg/kg), in combination with other sedating medications, is frequently employed. However, we have observed a decline in the use of chloral hydrate, which is appropriate considering its narrow therapeutic index and the lack of an antidote for toxicity (87).

### 9.9. Pentobarbital

Pentobarbital is a barbiturate that does not possess any inherent analgesic properties, but induces deep sedation, hypnosis, amnesia, and anticonvulsant activity in a dose-dependent manner. When administered intravenously, sedation becomes noticeable in 3–5 min and persists for approximately 30–40 min (88). However, the duration of sedation with pentobarbital can be prolonged, which makes it a less feasible option for high-volume outpatient pediatric dental services that depend on swift patient turnover (89).

### 9.10. Hydroxyzine

Hydroxyzine is a psychosedative medication that exerts antihistaminic, antiemetic, and antispasmodic effects. Available as hydroxyzine hydrochloride or hydroxyzine pamoate, this drug has a wide safety margin and is frequently used in pediatric conscious sedation. It can be administered as a sole agent or in conjunction with other medications such as midazolam. However, when used concurrently with other central nervous system depressants, hydroxyzine can increase the depressant effects. Several pediatric sedation studies have utilized doses ranging from 1 to 2 mg/kg when combined with other sedative medications (90). The sedative effect of hydroxyzine may appear somewhat delayed, but it endures for a sufficient duration, making it suitable for lengthy dental procedures.

### 9.11. Sevoflurane

Sevoflurane, a fluorinated methyl-propyl ether, functions as an inhaled anesthetic that acts on the gamma-aminobutyric acid (GABA)-A receptor. It is a reliable anesthetic medication that has a rapid onset and recovery time. Additionally, it offers a quick adjustment of anesthetic depth and has a high safety profile concerning the cardiovascular system. One advantage of using sevoflurane for sedation, particularly in pediatric patients with needle phobia or intellectual disabilities who are unable to cooperate with venous catheterization, is that it is easy to administer compared to sedation using intravenous drug injection. Due to these benefits, there is an expectation that the demand for sedation in dental treatment using sevoflurane will continue to grow (91). When carrying out dental procedures on pediatric or disabled patients, sevoflurane sedation is a more cost-effective option compared to general anesthesia because of its quicker induction and recovery times. However, sevoflurane sedation does have some drawbacks. The distinctive odor of the sedative may be challenging to tolerate for some patients, and there may be a need to secure the airway in cases of excessive sedation. Additionally, the anesthetic gas may spread to the treatment room (92).

### 9.12. Melatonin

Melatonin is an indoleamine that functions as an effective oral sleep aid. Its primary function is to modulate the circadian rhythm of sleep. Melatonin has been shown to have optimal efficacy as an initial anxiolytic agent for pediatric patients scheduled for surgical procedures (93). However, the efficacy of melatonin and the appropriate dosage for children have yet to be clearly defined. The optimal dose of 0.5 mg/kg was established for melatonin based on earlier reports (94). Ansari et al.’s research indicates that premedication with oral midazolam in pediatric patients is superior to that with melatonin, with higher levels of satisfaction reported by both parents and operators (95).

### 9.13. Reversal agents

Reversal drugs should not be administered routinely, but rather should be reserved for instances of oversedation or respiratory depression that persist beyond a transient period and when the patient fails to respond to verbal or tactile stimulation (23).

#### 9.13.1. Naloxone

Naloxone is an opioid-receptor antagonist used to treat opioid overdose and reverse the respiratory and central nervous system depressant effects of opioids (96). It is available in both parenteral and intranasal formulations, and has a relatively rapid onset of action (approximately 2 min) with a duration of approximately 20–40 min (97).

#### 9.13.2. Flumazenil

Flumazenil is a benzodiazepine reversal agent that competes with benzodiazepines for receptor sites through competitive inhibition. It is used to reverse central nervous system and respiratory depressant effects and decrease recovery time. Flumazenil possesses a short half-life, resulting in a short duration of action. It is important not to hesitate to use flumazenil if you are having difficulty getting patients to respond to verbal commands or if constant physiological monitoring indicates a trend toward non-manageable oxygen desaturation (98) (Table 4).

**Table 4:** Properties of procedural sedation agents used in pediatrics.

Medications	Dose	Onset time	Duration
Nitrous oxide	Inhaled starting at 100% O_2_ and increasing concentration nitrous oxide to effect.	30 s (peak effect 3 to 5 min)	3–5 min
Midazolam	PO 0.25-1 mg/kg (maximum dose 15 mg)	15–20 min	30–50 min
	IN 0.3–0.5 mg/kg (maximum dose 10 mg)	10–15 min	30 min
	IV 0.05–0.1 mg/kg (0.5–5 years) (maximum0.6 mg/kg)	2–3 min	30–50 min
	IV 0.025–0.05 mg/kg (6–12 years) (maximum 0.4 mg/kg)	10–20 min	60–120 min
	IM 0.1–0.5 mg/kg		
Ketamine	IV 1 mg/kg, given over 1 min; repeat dose (0.5 mg/kg) every 10 min as needed	1 min	15 min
	IM4mg/kg; repeat dose (2 mg/kg) after 10 min if needed	3–5 min	15–30 min
Dexmedetomidine	IN 1-4ug/kg (max 100ug)	15–30 min	55–100 min
	IV 1–3 ug/kg loading dose (max 100ug) over 10 min, followed by 0.5–1 ug/kg/h continuous infusion	5–10 min	15 min
Fentanyl	IV 1–1.5 mcg/kg as initial dose and titrated 1 mcg/kg every 3 min	1–3 min	30–60 min
	Maximum dose 4 mcg/kg		
	IN 1–3 mcg/kg	10 min	20 min
Propofol	IV 1–3 mg/kg; may be repeated at half-doses as needed	15–30 s	5–15 min
Etomidate	IV 0.2–0.3 mg/kg	1 min	5–15 min
Chloral hydrate	PO 25–100 mg/kg, after 30 min can repeat 25–50 mg/kg. Maximum dose:2 g or 100 mg/kg	15–30 min	60–120 min
Pentobarbital	IV 1–6 mg/kg, titrated in 1–2 mg/kg increments every 3–5 min to desired effect	3–5 min	30–40 min
	IM 2–6 mg/kg, maximum 100 mg	10–15 min	60–120 min
	PO 3–6 mg/kg maximum 100 mg(<4 years)	15–60 min	60–240 min
	PO 1.5-3 mg/kg, maximum 100 mg (>4 years)		
Naloxone	IV or IM 0.1 mg/kg/dose up to maximum of 2 mg/dose, may repeat every 2 min as needed	2 min	20–40 min
Flumazenil	IV 0.02 mg/kg/dose, may repeat every 1 min up to 1 mg	1–2 min	30–60 min

PO, per os (buccal); IV, intravenous; IM, intramuscular; IN, intranasal.

### 9.14. New innovation in drug development

Recent and evolving drug innovations are primarily focused on modifying the chemical structures of existing drugs or drug classes with the intention of improving their pharmacodynamic, pharmacokinetic, and side effect properties (82).

#### 9.14.1. Remimazolam

Remimazolam is a rapidly metabolized intravenously administered benzodiazepine sedative that induces sedation by binding to specific neurotransmitter receptors in the brain (99). It like remifentanil, has organ-independent elimination and acts on the same receptor as midazolam - γ-aminobutyric acid. As such, remimazolam is classified as a “soft drug,” which has been investigated for the creation of fast-acting sedatives with predictable recovery (100). Randomized controlled trials of procedural sedation have shown that remimazolam has a quicker onset and offset of hypnotic effect than midazolam. Remimazolam exhibits the cardiorespiratory stability typical of benzodiazepines, and its effects can be fully reversed by flumazenil (101). Remimazolam does not produce injection site pain, which is a common side effect in propofol use (observed in 18.7% of cases) (102). The incidence of intraoperative hypotension events is lower with remimazolam (22%) compared to propofol (49.3%) (102). There is no requirement for unscheduled mechanical ventilation when administering procedural sedation with the use of remimazolam (103). Propofol sedation has limitations such as pain at the injection site and potential respiratory and hemodynamic depression without a reversal agent. In contrast, remimazolam does not cause injection site pain and has a reversal agent, which makes it a potential candidate for primary sedation medication in pediatric sedation in the future (104).

The continuous infusion of remimazolam could prove to be a valuable sedative option during dental procedures. Regarding general anesthesia in adults, the recommended induction dose of remimazolam to achieve unconsciousness is 12 mg/kg/h, while a maintenance dose of 1 mg/kg/h is utilized in Japan and South Korea (105). In the United States, a recommended dose of 5 mg of remimazolam administered *via* an IV push injection over 1 min is suggested for inducing procedural sedation. If necessary, additional IV doses of remimazolam of 2.5 mg over 15 s may be given with a minimum interval of 2 min between doses. In the European Union, for patients not receiving a concurrent opioid, an initial dose of 7 mg of remimazolam is recommended for inducing procedural sedation (106).

Despite remimazolam appearing to be an excellent sedative, only a limited number of studies have evaluated its use for sedation in the pediatric population. In order to enhance patient safety and comfort during dental procedures, further studies on the use of remimazolam for dental sedation in pediatric patients are necessary.

#### 9.14.2. ADV6209

ADV6209, which has received approval as a pediatric anxiolytic in Europe, could potentially replace midazolam. One of the benefits of ADV6209 is that it is a 0.2% aqueous midazolam formulation combined with a gamma-cyclodextrin complex that masks the bitter taste and improves solubility, with the addition of sucralose and orange aroma (107). Over 75% of the drug is absorbed within 30 min of oral administration, and in adults, it has a half-life of 2.66 h and a duration of 48.5 min (17).

#### 9.14.3. ABP-700

Cyclopropyl-methoxycarbonyl metomidate, also known as ABP-700, is a second-generation etomidate that binds to the same site on the GABAA receptor as etomidate. ABP-700 is designed with an ester bond that undergoes rapid hydrolysis in the body by non-specific tissue esterases, producing an inactive carboxylic acid metabolite. This potent anesthetic agent has minimal hemodynamic effects and adrenal suppression in animal studies. ABP-700 is a novel, potent, positive allosteric modulator of the GABAA receptor and is currently being developed for general anesthesia and procedural sedation (85).

### 9.15. Multi-drug delivery for pediatric dental sedation

Acknowledging that a singular pharmaceutical agent does not provide optimal sedative outcomes, it is customary for pediatric dental professionals to amalgamate various medications. This polypharmacy technique offers cumulative, mutually augmenting, and intensified sedative effects, thereby allowing for decreased dosages of each individual medication. Furthermore, medicines can be combined to introduce effects that are not innately present in a single agent (40). However, an analysis of case reports in the United States concerning severe neurological impairment and fatalities revealed that these accidents were caused by the combination of more than three medications, excessive dosages, and insufficient training (89). Limited research, with available studies using mixed methodological approaches, has made it difficult to judge either regimen as being superior to the other (108).

#### 9.15.1. Propofol+ketamine

Ketamine poses the potential risks of undesired adverse effects such as emergence phenomenon, vomiting, and laryngospasm. However, these unfavorable events can be mitigated by combining ketamine with propofol. Propofol effectively mitigates the emetogenic and psycho-cognitive effects of ketamine, while the combined effect of ketamine decreases the likelihood of propofol-induced respiratory depression and hypotension (109).

#### 9.15.2. Midazolam+ketamine

Studies have indicated that premedication regimens combining the anxiolytic properties of midazolam with the analgesic properties of ketamine resulted in superior pediatric behavior compared to the administration of these drugs separately (110).

#### 9.15.3. Midazolam+fentanyl

The combination of fentanyl and midazolam is a commonly employed regimen for procedural sedation and analgesia in pediatric patients, with a robust safety profile when both drugs are meticulously titrated to effect. Fentanyl yields desirable effects, including analgesia, sedation, enhanced mood, and extended duration of action, which are not typically observed with other frequently utilized sedatives. Moreover, opioids possess the potential benefit of decreasing the incidence of disinhibitory paradoxical reactions. Co-administration of an opioid alongside a benzodiazepine appears to reduce the frequency of restlessness and agitation, which are more commonly encountered with high doses of benzodiazepines (40).

#### 9.15.4. DEX+ketamine

Although procedural sedation using dexmedetomidine is generally safe, bradycardia caused by this medication could be a potential issue. However, it is worth noting that ketamine has a unique ability to stimulate the cardiovascular system. Therefore, combining low doses of ketamine with dexmedetomidine could lead to a more stable cardiorespiratory profile (111).

## 10. Fasting

The need for fasting prior to PSA is a controversial topic. Current ASA guidelines for fasting prior to PSA recommend 2-h clear,4-h breast milk,6-h formula, and 8-h solids. However, there is little evidence that this approach actually prevents aspiration. The updated Practice Guidelines for Moderate Procedural Sedation and Analgesia, released in 2018, recommends a slightly different approach. According to these guidelines, patients should fast for 2 h if they have had clear liquids, 4 h if they have had breast milk, 6 h if they have had formula, and 6 h if they have had ‘light foods’ (112). Lowering the fasting time for clear liquids can improve the patient’s experience by reducing the duration of fasting. According to the updated 2023 ASA guidelines on fasting, include the use of oral midazolam, it is recommended to minimize fasting duration in children. Therefore, every effort should be made to permit clear liquids in healthy children up to 2 h before medical procedures (113), and that early eating is safe as long as the patients have recovered from anesthesia and swallowing function evaluation is done (114). It’s important to note that following the ASA fasting guidelines is recommended for patients undergoing moderate sedation, where the child may not be able to maintain verbal contact or may undergo deep sedation. The use of nitrous oxide for sedation does not require fasting.

## 11. Monitoring

Over the last three decades, sedation has become a commonly used alternative to general anesthesia. However, it is worth noting that almost 80% of sedation-related emergencies initially present as respiratory compromise (89). As the level of sedation deepens, the airway protective reflex decreases, and the likelihood of airway obstruction or foreign body aspiration increases. Therefore, appropriate respiratory monitoring and airway management are essential during sedation procedures. Various organizations, including the Joint Commission on Accreditation of Healthcare Organizations (JCAHO), the American Society of Anesthesiologists (ASA), the American Academy of Pediatrics (AAP), and the American Academy of Pediatric Dentistry (AAPD), have published guidelines aimed at reducing the risks associated with sedation in children and ensuring safe patient monitoring. These guidelines are mostly consistent and follow the principles set out by the ASA. All guidelines for respiratory function monitoring recommend the following (115):

1. Continuous monitoring of oxygenation through pulse oximetry is necessary.
2. Ventilation should Be monitored periodically during moderate sedation and continuously during deep sedation and general anesthesia.

Monitoring equipment typically includes cardiac, blood pressure, pulse oximetry, and respiratory monitors. The use of an EtCO2 monitor is highly desirable.

Continual evaluation of the extent of sedation is of paramount importance in detecting the patient’s transition into profound sedation and the concomitant risk of impaired protective reflexes. The guidelines established by the American Academy of Pediatrics (AAPD) and the American Society of Anesthesiologists (ASA) dictate that the depth of sedation be persistently monitored throughout the procedure. In this regard, the BIS monitor can furnish an additional, objective criterion for measuring the depth of sedation and thereby enhancing patient safety. It gathers processed EEG parameters to provide a numeric measure of the hypnotic effect of anesthetic or sedative drugs on brain activity. The utility of the BIS monitor during general anesthesia has been validated in multiple pediatric studies.

It is crucial for the clinical team to be able to identify signs of a deteriorating patient and respond appropriately. While monitoring equipment is essential, there is no single piece of equipment that can replace the role of a capable and vigilant sedation provider who is responsible for monitoring the patient during the sedation procedure (Table 5).

**Table 5:** Necessary equipment for safe administration of pediatric sedation “SOAPME.”

S = Size	Appropriate suction catheters and a functioning suction apparatus
O = Oxygen	An adequate oxygen supply and flow meters or devices to allow for delivery
A = Airway	Size-appropriate bag-valve-mask, nasopharyngeal and oropharyngeal airways, laryngeal-mask-airway, laryngoscope blades, endotracheal tubes and stylet, face mask
P = Pharmacy	All basic drugs needed for life support during an emergency, including antagonists
M = Monitors	Functioning pulse oximeters, size-appropriate oximeter probes, end-tidal carbon dioxide monitor, other monitors as appropriate for the procedure
E = Equipment	Special equipment or drugs as needed

From “Pediatric Procedural Sedation, Analgesia, and Anxiolysis” by B. Klick, A. Serrette, and J.M. Clingenpeel, 2017, Emergency Medicine, 49, pp. 352–362.

## 12. Conclusion

Pediatric dental providers should exercise caution in case selection and customize the route, medication, and dosage based on the patient and procedure. Patient safety should be the top priority, and providers should adhere to established best practices for sedation. The key to safe sedation lies in the early detection and management of potential adverse events. The continued development and safety of pediatric sedation will depend on a thorough pre-sedation assessment and a willingness to explore both traditional and new sedatives, either alone or in combination. Several important questions remain unanswered, such as the potential benefits and risks of using combination sedatives during a sedation procedure and their impact on neurocognitive outcomes.

## Author contributions

FG: conceptualization, visualization, writing—original draft, and polishing the manuscript. YW: conceptualization, funding acquisition, and writing—last draft. All authors contributed to the article and approved the submitted version.

## Funding

The present study was supported by Chongqing Medical University 2022 Future Medical Youth Innovation Team Development Support Program (grant: w0147).

## Conflict of interest

The authors declare that the research was conducted in the absence of any commercial or financial relationships that could be construed as a potential conflict of interest.

## Publisher’s note

All claims expressed in this article are solely those of the authors and do not necessarily represent those of their affiliated organizations, or those of the publisher, the editors and the reviewers. Any product that may be evaluated in this article, or claim that may be made by its manufacturer, is not guaranteed or endorsed by the publisher.
